# The 2023 European Task Force Criteria for Diagnosis of Arrhythmogenic Cardiomyopathy: Historical Background and Review of Main Changes

**DOI:** 10.31083/j.rcm2509348

**Published:** 2024-09-24

**Authors:** Francesca Graziano, Alessandro Zorzi, Simone Ungaro, Barbara Bauce, Ilaria Rigato, Alberto Cipriani, Martina Perazzolo Marra, Kalliopi Pilichou, Cristina Basso, Domenico Corrado

**Affiliations:** ^1^Department of Cardiac, Thoracic and Vascular Sciences and Public Health, University of Padova, 35128 Padova, Italy; ^2^Department of Sports Medicine, Semmelweis University, 1085 Budapest, Hungary

**Keywords:** arrhythmogenic cardiomyopathy, cardiac magnetic resonance, diagnosis, ventricular arrhythmia, sudden death

## Abstract

Arrhythmogenic cardiomyopathy (ACM) is a cardiac disease featured by non-ischemic myocardial scarring linked to ventricular electrical instability. As there is no single gold-standard test, diagnosing ACM remains challenging and a combination of specific criteria is needed. The diagnostic criteria were first defined and widespread in 1994 and then revised in 2010, approaching and focusing primarily on right ventricular involvement without considering any kind of left ventricular variant or phenotype. Years later, in 2020, with the purpose of overcoming previous limitations, the Padua Criteria were introduced by an international expert report. The main novel elements were the introduction of specific criteria for left ventricular variants as well as the use of cardiac magnetic resonance for tissue characterization and scar detection. The last modifications and refinement of these criteria were published at the end of 2023 as the European Task Force criteria, by a “head-quarter” of ACM international experts, proving the emerging relevance of this condition besides its difficult diagnosis. In this review, emphasizing the progress in understanding the aetiology of the cardiomyopathy, an analysis of the new criteria is presented. The introduction of the term “scarring/arrhythmogenic cardiomyopathy” sets an important milestone in this field, underlying how non-ischemic myocardial scarring—typical of ACM—and arrhythmic susceptibility could be the main pillars of numerous different phenotypic variants regardless of etiology.

## 1. Introduction

Arrhythmogenic cardiomyopathy (ACM) is a heart condition characterized by the gradual substitution of ventricular myocardium with fibro-fatty tissue [1]. This disorder increases the risk of life-threatening ventricular arrhythmias (VAs) and sudden cardiac death (SCD) [2].

The earliest documented case of ACM as a heredo-familial disease dates back to 1736, when Giovanni Maria Lancisi [3] reported its recurrence within a family. Initially, it was believed that ACM exclusively affected the right ventricle (RV). In 1982, Marcus *et al*. [4] introduced the term “arrhythmogenic right ventricular dysplasia” after studying 24 adult patients who exhibited ventricular arrhythmias with a left bundle branch block (LBBB) pattern, suggesting an origin in the affected RV. It was perceived as a developmental anomaly of the RV muscle tissue at the time. Later on, for the first time Thiene and colleagues [5] identified ACM as a primary cause of SCD in young individuals and athletes. Post-mortem examinations indicated a myocardial disorder occurring after birth, as evidenced by histopathological findings revealing areas of inflammation, deterioration, and tissue death, leading to gradual myocardial loss [5]. The term dysplasia was discarded in favor of “arrhythmogenic right ventricular cardiomyopathy” (ARVC) with the identification of gene defects linked to desmosomal proteins. Consequently, ACM was incorporated into the World Health Organization (WHO) nomenclature and cardiomyopathy classification [6].

## 2. Evolution of Diagnostic Criteria: A Historical Overview

The diagnostic path of ARVC started in 1994 when a Task Force (TF) composed of experienced clinicians specialized in cardiomyopathies estabilished appropriate diagnostic criteria for the first time, aiming for a common and shared gold standard. The original criteria were based on multiple frameworks unified into six different sections: (1) global or regional dysfunction and imaging-detected structural alterations in RV; (2) tissue characterization via endomyocardial biopsy (EMB); (3) electrocardiogram (ECG) repolarization abnormalities; (4) ECG depolarization abnormalities; (5) arrhythmias; (6) family history. The single criteria were considered “major” or “minor” according to their specificity for discerning between ARVC and other heart diseases with a similar clinical presentation, mainly idiopathic right ventricular outflow tract (RVOT) ventricular tachycardia (VT) or biventricular dilated cardiomyopathy (DCM). The diagnostic algorithm proposed in 1994 set the necessity of either 2 major criteria or 1 major plus 2 minor criteria, or 4 minor criteria, all from different categories [7].

Favoring specificity, 1994 TF criteria lacked sensitivity for asymptomatic patients and initial phases of the disease. Original criteria focused mainly on clinicians on-field expertise, primarily facing severe and advanced stages of ARVC.

Contextually to progressive evolution of ACM knowledge, in 2010 the revised International Task Force (ITF) criteria were produced with the aim of improving such limitations. Based on the same classification of the previous, the updated version showed a diagnostic accuracy enhancement, taking advantage of precise quantitative imaging parameters to define different degrees of structural and functional abnormality compared to normal RV characteristics [8]. The 2010 criteria extended the diagnostic range identifying two new phenotypes, namely “borderline” and “possible” which fulfill specific major/minor criteria. For the first time, the definition of fibrofatty myocardial replacement were introduced alongside to EMB weight in terms of significance. Furthermore, molecular genetic information was included in the family history category highlighting the relevance of inheritance.

In the following years, a progressive expansion of knowledge and diagnostic skill, allowed recognizing that ACM often involves both ventricles, with a prevalence of either the right or the left one and, in some cases, with exclusive left ventricular (LV) involvement. This permitted the replacement of the concept of “arrhythmogenic right ventricular cardiomyopathy” to the broader term of “arrhythmogenic cardiomyopathy”.

In relation to this improvement, in 2019, an international group of experts in cardiomyopathies critically reviewed the 2010 criteria, underlining three main areas for improvement: (1) the need to increase the role of cardiac magnetic resonance (CMR), not just for morpho-functional assessment but also for tissue characterization [9]; (2) the need for specific diagnostic criteria for LV involvement, criticizing the inadequate identification of an appropriate cases of LV-ACM patients; (3) the peculiarity of considering genetic testing as a major criterion enabling diagnosing the disease even when there is a lack of morphological and functional ventricular alterations (as opposed to other cardiac diseases in which the diagnosis always requires demonstration of clinical abnormalities and genetic testing is used for confirmation) [10]. In addition to these observations, experts emphasized the need for revising specific RV criteria.

During the same year, a Heart Rhythm Society (HRS) Consensus expert panel published a statement covering general and crucial aspects, genetics, pathological mechanisms, and diagnosis of cardiomyopathies, giving a useful and unique instrument to evaluate, treat and manage ACM [11].

On this basis, in 2020 a group of physicians from the University of Padua, with the help of international colleagues, proposed new criteria for the diagnosis of ACM: the so-called “Padua Criteria” [12]. In the following two years, this Consensus was then extended to a TF of European experts in different aspects of ACM (clinical, imaging, electrophysiology and genetics) and, in 2022, a consensus conference was held in Florence with the aim to revise and upgrade the Padua criteria. The results of the conference were published the following year [13].

In addition to some modifications of the diagnostic criteria, an important aspect of the disease was highlighted: myocardial “scarring” is the distinctive pathological feature of the disease, resulting from myocyte apoptosis/necrosis and subsequent fibro-fatty “repairing” [14]. Myocardial scar is the substrate of life-arrhythmic arrhythmias such as VT/ventricular fibrillation (VF) and can lead to a progressive decline in systolic function [5, 10, 14]. Highlighting this concept, a new, more inclusive denomination of the disease is proposed in the document: “scarring/arrhythmogenic cardiomyopathy”. This remarks again the central role of the histopathological hallmark, consistently identifiable across all the ACM phenotypes regardless of the underlying etiology.

## 3. The European Task Force Diagnostic Criteria

Similar to the Padua Criteria, the new European TF diagnostic criteria distinguish three consequent steps in the ACM diagnostic process: (1) ventricular involvement; (2) phenotypic definition; (3) etiology and classification.

### 3.1 First Step: Criteria for RV and LV Involvement

The 2023 European Task Force diagnostic criteria maintain the same “six-category structure” as the previous ones and focus on the specific involvement of right, left or both ventricles using a multiple step diagnosis process [15].

The first step, based on dominant ventricular involvement, allows to identify three primary phenotypic variants: (1) *Arrhythmogenic right ventricular cardiomyopathy* (ARVC), the classical and “historical” phenotype primarily affecting the RV with no apparently detectable LV structural or morpho-functional abnormalities; (2) *Arrhythmogenic biventricular cardiomyopathy* (Biv-ACM) phenotype, which requires demonstration of morpho—functional and/or structural abnormalities of both RV and LV; (3) *Arrhythmogenic left ventricular cardiomyopathy* (ALVC), characterized by isolated LV abnormalities. Following this notable right and left distinction, the 2023 criteria consist of two different blocks to distinguish and identify clinical, electrocardiographic, and imaging parameters of left or right ventricular involvement, independently.

As the previous 2020 Padua Criteria, distinction between “major” or “minor” criterion is maintained and based on their diagnosis specificity (Table 1, Ref. [13]).

**Table 1.** **European Task Force criteria for diagnosis of arrhythmogenic cardiomyopathy**.

Category	Criteria for RV involvement	Criteria for LV involvement
1. Morpho-functional ventricular abnormalities	*Major*	*Minor*
	• Regional RV akinesia, dyskinesia, or aneurysm plus one of the following:	• Global LV systolic dysfunction, with or without LV dilatation (increase of LV EDV according to the imaging test specific nomograms for age, sex, and BSA)
	- global RV dilatation (increase of RV EDV according to the imaging test specific nomograms for age, sex and BSA)	
	*or*	
	- global RV systolic dysfunction (reduction of RV EF according to the imaging test specific nomograms for age and sex)	
	*Minor*	
	• Regional RV akinesia, dyskinesia or aneurysm of RV free wall	
2. Structural myocardial abnormalities	*Major*	*Major*
	• Fibrous replacement of the myocardium in ≥1 sample, with or without fatty tissue, at histology	• “Ring-like” LV LGE (subepicardial or midmyocardial stria pattern) of ≥3 segments (confirmed in 2 orthogonal views)
	*Minor*	*Minor*
	• Unequivocal RV LGE (confirmed in 2 orthogonal views) in ≥1 RV region(s) (excluding tricuspid valve)	• LV LGE (subepicardial or midmyocardial stria pattern) of 1 or 2 Bull’s Eye segment(s) (in 2 orthogonal views) of the free wall, septum, or both (excluding patchy, focal or septal junctional LGE)
3. ECG repolarization abnormalities	*Major*	*Minor*
	• Negative T waves in right precordial leads (V1, V2, and V3) or beyond in individuals ≥14-year-old (in the absence of complete RBBB and not preceded by J-point/ST-segment elevation)	• Negative T waves in left precordial leads (V4–V6) (in the absence of complete LBBB)
	*Minor*	
	• Negative T waves in leads V1 and V2 in males ≥14-year-old (in the absence of RBBB and not preceded by J-point/ST-segment elevation)	
	• Negative T waves beyond V3 in the presence of complete RBBB	
	• Negative T waves beyond V3 in individuals <14-year-old	
4. ECG depolarization and conduction abnormalities	*Minor*	*Major*
	• Epsilon wave (reproducible low-amplitude signals between end of QRS complex to onset of the T wave) in the right precordial leads (V1 to V3)	• Low QRS voltages (<0.5 mV peak to peak) in all limbs leads in the absence of other causes (e.g., cardiac amyloidosis, obesity, emphysema, or pericardial effusion)
	• Terminal activation duration of QRS ≥55 ms measured from the nadir of the S wave to the end of the QRS, including R’, in V1, V2, or V3 (in the absence of complete RBBB)	
5. Arrhythmias	*Major*	*Minor*
	• Frequent ventricular extrasystoles (>500 per 24 h), non-sustained or sustained ventricular tachycardia of LBBB morphology with non-inferior axis	• Frequent (>500 per 24 h) or exercise-induced ventricular extrasystoles with a RBBB morphology or multiple RBBB morphologies (excluding the “fascicular pattern”)
	*Minor*	• Non-sustained or sustained ventricular tachycardia with a RBBB morphology (excluding the “fascicular pattern”)
	• Frequent ventricular extrasystoles (>500 per 24 h), non-sustained or sustained ventricular tachycardia of LBBB morphology with inferior axis (“RVOT pattern”)	• History of cardiac arrest due to ventricular fibrillation or sustained ventricular tachycardia of unknown morphology
	• History of cardiac arrest due to ventricular fibrillation or sustained ventricular tachycardia of unknown morphology	
6. Family history/genetics	*Major*	
	• Identification of a pathogenic ACM-gene variant in the patient under evaluation	
	• ACM confirmed in a first-degree relative who meets diagnostic criteria	
	• ACM confirmed pathologically at autopsy or surgery in a first-degree relative	
	*Minor*	
	• Identification of a likely-pathogenic ACM-gene variant in the patient under evaluation	
	• History of ACM in a first-degree relative in whom it is not possible or practical to determine whether the family member meets diagnostic criteria	
	• Premature sudden death (<35 years of age) due to suspected ACM in a first-degree relative	
	• ACM confirmed pathologically or by diagnostic criteria in second-degree relative	

ACM, arrhythmogenic cardiomyopathy; BSA, body surface area; ECG, electrocardiogram; EDV, end diastolic volume; EF, ejection fraction; LBBB, left bundle branch block; LGE, late gadolinium enhancement; LV, left ventricle; RBBB, right bundle branch block; RV, right ventricle; RVOT, right ventricular outflow tract. Adapted from [13].

Diagnostic criteria are divided in these category parameters: (1) *Morpho-functional ventricular abnormalities*; (2) *Structural myocardial abnormalities*; (3) *ECG repolarization abnormalities*; (4) *ECG depolarization/conduction abnormalities*; (5) *Arrhythmias*; (6) *Genetics and family history*.

#### 3.1.1 Morpho-Functional Ventricular Abnormalities

Morpho-functional abnormalities can be detected using various imaging techniques such as echocardiography, CMR, multidetector computed tomography (MDCT) and ventricular angiography, often used when CMR is impractical due to incompatible implantable cardioverter-defibrillator (ICD), frequent arrhythmias or even claustrophobia and patients’ other personal reasons [16].

Echocardiography is often the preferred initial imaging modality due to its widespread availability, non-invasiveness, and repeatability, providing valuable insights into the cardiac phenotype, disease etiology, morphology, hemodynamics, and severity. However, in cases of suspected ACM, it is crucial not to overlook the infero-basal (sub-tricuspid) RV region, which is commonly affected but may be neglected in standard echocardiographic views. Thus, obtaining an off-axis 2-chamber apical view focused on assessing the inferior RV wall is advisable [17].

To enhance diagnostic accuracy and specificity, the primary morpho-functional criterion for the RV necessitates the presence of global RV enlargement or systolic dysfunction, accompanied by regional wall motion irregularities like akinesia, dyskinesia, or aneurysm. Utilizing up-to-date reference values for cardiac chamber dimensions and function, adjusted for factors such as gender, age, body surface area, and specific considerations for athletes, is recommended [18, 19].

Moreover, a minor criterion includes regional wall motion abnormalities even without RV dilation or dysfunction, recognizing the localized nature of ACM and its impact on segmental contractility. However, caution is warranted in interpreting such abnormalities, particularly in CMR, where non-pathological wall motion abnormalities may lead to misinterpretation [20, 21, 22]. The morpho-functional criterion for the LV (ejection fraction reduction with or without dilation) is considered minor due to its limited specificity in diagnosing left-sided ACM variants versus other LV diseases. This designation is due to the similarity of LV abnormalities with conditions like ischemic heart disease. Notably, ventricular remodeling in ALVC is often detected through echocardiography or cine-CMR, revealing a hypokinetic and non-dilated (or mildly dilated) LV [20].

#### 3.1.2 Structural Myocardial Abnormalities

CMR and EMB are used to detect characteristic fibro-fatty or fibrous myocardial-tissue found in ACM. Nowadays, CMR has a central role in identifying RV late gadolinium enhancement (LGE), although it is widely limited due to current technological limitations, like sub-optimal spectral resolution and an inadequate contrast-to-noise ratio in front of the thin RV wall quantification. The most effective specificity is obtained through evaluating changes in wall motion alongside abnormalities in tissue characterization. Consequently, the identification of LGE in at least one region of RV CMR imaging has been designated as a minor criterion for RV involvement [13]. Specific LV LGE, predicting myocardial scar, reveals itself soon in ACM, anticipating visible wall motion alterations. LGE shows a distinct appearance typically in the subepicardial or, occasionally, in the mid-myocardial layers of the LV free wall, mainly in the inferolateral region. Confirming the presence of LGE is crucial, necessitating verification in two orthogonal planes or utilizing 3-dimensional (3D)-LGE imaging to mitigate potential artifacts. Due to its high specificity, LV LGE involving ≥3 segments at the short axis Bull’s Eye, either contiguous in the same slice with a “ring-like” pattern or discontinuous, is considered a major criterion. Segmental LV LGE affecting 1 or 2 LV Bull’s Eye segments is classified as minor. Patchy or focal LV LGE is intended as non-diagnostic and lacks clinical relevance in the absence of other abnormal findings. It is essential to note that “septal junctional” LGE at RV insertion points is not indicative of ACM due to its non-pathological significance [20, 23, 24]. It is important to understand that, while the finding of fatty tissue alone is not considered a diagnostic criterion, its identification using CMR or MDCT dedicated sequences in regions of LGE/scar strengthen diagnostic specificity [21].

Due to its invasive nature and associated risks, EMB is selectively advised when the diagnosis or exclusion of ACM depends on histological evidence of replacement-type fibrosis, in fatty tissue presence or not and is found among major structural criteria. EMB becomes particularly crucial in identifying non-genetic variants of ACM, such as isolated cardiac sarcoidosis, where the diagnosis is based on histological evidence of the typical noncaseating epithelioid cell granulomas in the myocardium [25, 26].

Electro-anatomic voltage mapping, despite not usually being recommended for diagnosis, may be used to enhance sensitivity for RV scars in selected patients undergoing electrophysiological study and catheter ablation for sustained VT [22, 27].

#### 3.1.3 ECG Repolarization Abnormalities

Regarding RV involvement, as a major criterion there is the presence of T-wave inversion (TWI) in right precordial leads (V1–V3) or beyond, while the identification of TWI confined to V1–V2 leads only is a minor criterion. Both criteria apply to individuals older than 14 years old and requires the absence of complete right bundle branch block (RBBB) and, particularly in athletes, of J-point/ST-segment elevation. In fact, TWI preceded by J-point/ST-segment elevation is a variant of benign early repolarization. If complete RBBB is present, TWI beyond V3 is grouped into the minor criteria. As TWI in children is normal only in V1–V3, TWI beyond V3 is also considered a minor criterion for individuals younger than 14 [28].

The extension of TWI from V1 to lateral leads V4–V6 predicts a severe dilatation of the RV rather than a LV involvement [29]. LV specific involvement can be predicted when TWI does not include leads V1–V3 and is found only in left precordial leads (V4–V6) in the absence of complete LBBB. Due to the low specificity of this ECG finding, that may be found in several other cardiac diseases, it is considered a minor criterion [30].

#### 3.1.4 ECG Depolarization and Conduction Abnormalities

Signal average ECG (SAECG) values are no longer considered in the criteria, due to low diagnostic accuracy, lack of specificity and its difficult interpretation. Despite that, they can be useful for risk stratification: SAECG can be used as a non-invasive tool to detect the presence of late potentials, which are indicative of heterogeneous slow-conducting myocardium in which normal myocardium is replaced by fibrofatty tissue, contributing to the perpetuation of ventricular arrhythmias [31, 32].

Excluding RBBB, RV conduction abnormality indicators are now classified as minor criteria. Within these is included terminal activation duration (TAD) of the QRS ≥55 msec in right precordial leads (V1–V3), measured from the nadir of the S wave to the end of the QRS. The “notable” epsilon wave, defined by low-amplitude high-frequency signals between the end of the QRS complex and the onset of the T wave in right precordial leads, is also considered a minor criterion. However, its identification and interpretation are altered by ECG filtering and sampling rate, in addition to high variability between observers and experts [33].

Low QRS voltages in limb leads (peak-to-peak QRS amplitude <0.5 mV) usually indicates LV involvement. Progressive loss of LV myocardial mass with fibro-fatty replacement can lead to this reduction in electrical activity. This is included as an important major criterion if other potential causes of low QRS voltages such as emphysema, obesity, pericardial effusion, or inappropriate setting of low band-pass filters (<100 Hz) are excluded [34, 35].

#### 3.1.5 Arrhythmias

Regarded as the main ACM arrhythmic events, premature ventricular contractions (PVCs) typically emerge from scars and fibro-fatty replacement zones or close to these. PVCs are evaluated considering their absolute sum (>500 PVCs/24 h), morphology (on 12-lead ECG, 24-hour Holter monitoring or 12-lead ECG exercise test) and complexity. According to the European TF criteria, PVCs or VT with a LBBB/superior axis morphology originating from the RV free wall or interventricular septum are more peculiar for ACM, indeed they are included among major criteria. Otherwise, PVCs originating from the RVOT with LBBB/inferior axis morphology, are considered as less specific for ACM and are frequently idiopathic, constituting a minor criterion [36].

The detection of PVCs or VT exhibiting a well-defined RBBB morphology (wide and positive QRS in V1) suggesting the origin from the LV, represents a minor criterion for LV involvement if they are frequent or exercise-induced [37]. In patients with a LV scar involving the lateral or infero-lateral wall, the prevalent PVCs morphology is represented by RBBB/superior axis type with wide QRS complex in V1 lead, exhibiting a late precordial transition beyond V3 lead. This can be representative not only for ALVC but also for Biv-ACM, once again demonstrating the myocardial involvement of both ventricles [23]. Furthermore, among minor criteria for both RV and LV involvement, is a history of cardiac arrest resulting from VF or VT, even with unknown morphology of QRS.

#### 3.1.6 Family History/Genetics

Witnessing the variability in ACM presentation and ventricular involvement among relatives with the same genetic mutation, this category encompasses family history and molecular genetics applicable to both RV and LV assessments. These criteria aim to prevent misinterpretation of genetic results and misdiagnosis by offering specific guidelines for genotyping [38]. It is highly recommended to conduct genetic testing for individuals showing ACM symptoms, improving the screening of family members and early identification of gene carriers [39]. Major criteria include the identification of pathogenic ACM gene mutations (according to the American College of Medical Genetics (ACMG) 2015 classification [40]) in the proband and having a first-degree relative with a confirmed ACM diagnosis. Otherwise, minor criteria involve finding a likely-pathogenic gene mutation in the proband, suspecting ACM in a first-degree relative without confirmation, suspecting ACM in a first-degree relative who died suddenly before 35, and a confirmed ACM diagnosis in a second-degree relative.

### 3.2 Second Step: Phenotype Definition

The second stage in the diagnostic process involves identifying the ACM phenotype by evaluating the fulfillment of criteria related to both RV and LV involvement. Referring to the European TF criteria, any diagnosis of ACM requires at least one criterion, whether major or minor, from either the first - morpho-functional abnormalities - category or the second - structural abnormalities - category. This distinction is pivotal as ACM is fundamentally a structural heart disease. In other words, the absence of any manifestation of morpho-functional or structural abnormalities prevents an ACM diagnosis. These two categories delineate the phenotypic triple variants. If the morpho-functional and structural criteria are exclusively met for the RV, the potential diagnosis is ARVC; if they are fulfilled solely for the LV, ALVC is the likely diagnosis. Alternatively, if the criteria for both ventricles are fulfilled, the diagnosis may be Biv-ACM.

**Fig. 1.**
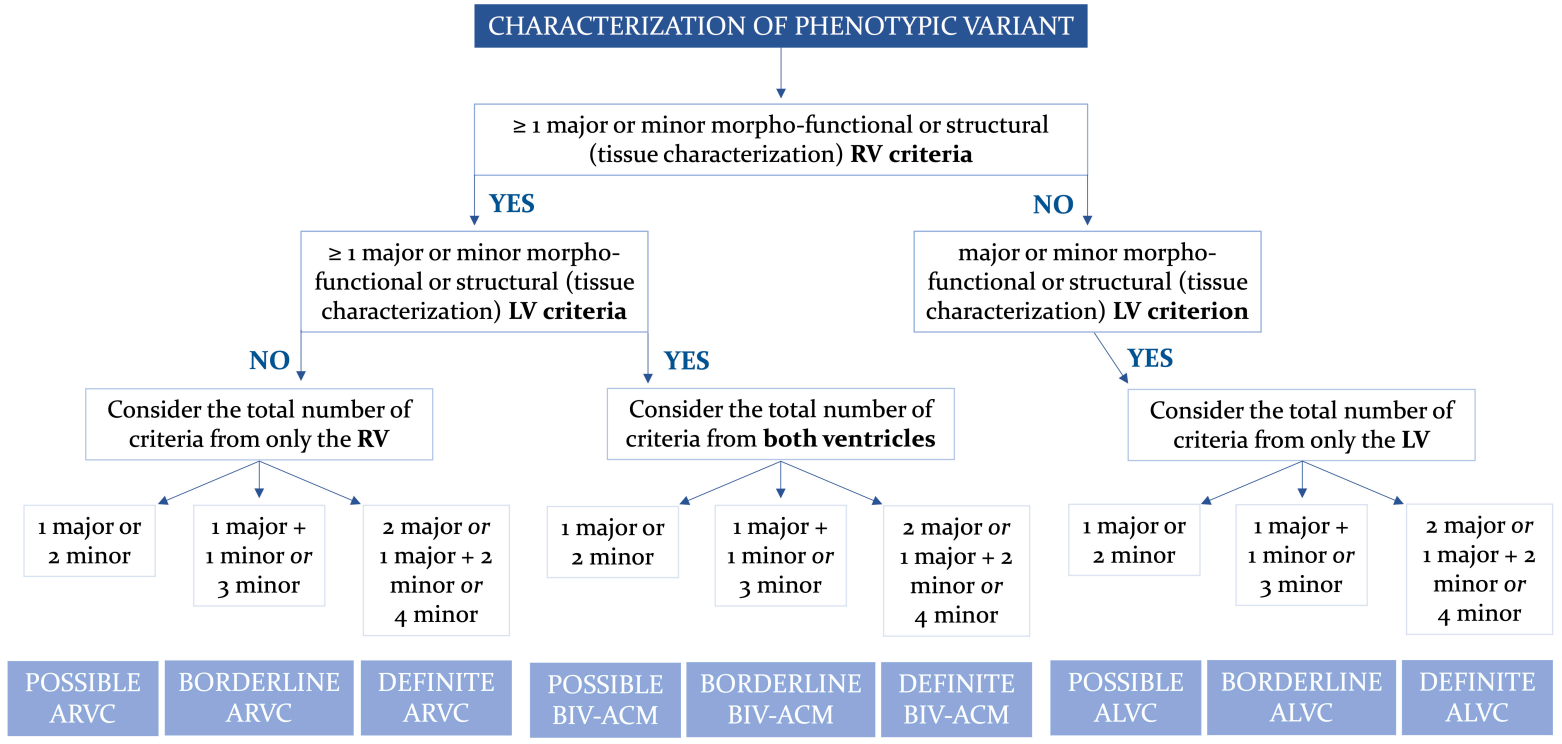
**Flowchart for phenotypic characterization of ACM**. Adapted from Corrado *et al*. [13]. ACM, arrhythmogenic cardiomyopathy; ALVC, arrhythmogenic left ventricular cardiomyopathy; ARVC, arrhythmogenic right ventricular cardiomyopathy; BIV-ACM, biventricular arrhythmogenic cardiomyopathy; LV, left ventricle; RV, right ventricle.

To estimate the probability of the disease, the other categories of criteria are then considered; only the criteria for ARVC can be considered in the presence of morpho-functional and/or structural abnormalities of only the RV; only the criteria for ALVC if only morpho-functional and/or structural LV involvement is present; criteria for both RV and LV involvement can be considered if both ventricles are affected by morpho-functional and/or structural changes. A “definite” diagnosis is confirmed if there are either 2 major criteria, or 1 major and 2 minor criteria, or 4 minor criteria; a “borderline” diagnosis if there are either 1 major and 1 minor criterion, or 3 minor criteria met; a “possible” diagnosis if there is either 1 major criterion or 2 minor criteria satisfied (Fig. 1, Ref. [13]) [41].

### 3.3 Third Step: Etiology and Classification

ACM is predominantly inherited as an autosomal dominant trait, showing variable expressivity and incomplete penetrance. The primary cause of inherited ACM, accounting for approximately 50% of cases, is represented by pathogenic variants in genes encoding desmosomal proteins playing a critical role in the electromechanical connection of cardiomyocytes and intracellular signaling. Although ACM, whether with RV or LV manifestations, is typically linked to these gene defects, variants originating from non-desmosomal genes encoding ion channels and cytoskeletal components (“genocopies”) exist, often associated with inherited neuromuscular disorders [22, 39, 42]. Despite its high specificity, diagnostic score can be achieved by other cardiac diseases that mimic ACM phenotype and known as “phenocopies”. Examples are cardiomyopathies associated with autoimmune multisystem diseases, cardiac sarcoidosis, and inflammatory cardiomyopathies, such as post-viral myocarditis. Differentiating between these etiological categories can be challenging especially after new emerging evidence suggests complex interactions between myocardial and inflammatory genetic factors [43]. It is essential to note that the existence of non-ischemic myocardial scars after a bout of overt acute myocarditis does not rule out a genetic origin. Indeed, in cases of ACM, inflammation might contribute to myocyte damage, resembling acute myocarditis-like episodes now acknowledged as “hot phase” [43].

Targeted clinical work-up, based on disease-specific tests and diagnostic criteria, is essential to identify and characterize the specific cause of ACM. This is crucial for determining ACM clinical outcomes, disease progression, involvement of multiple organ systems and the risk of SCD, as these factors possess high variability depending on the etiology. As with the past definitions and classifications, European TF underlines the presence of a notable portion of cases identifiable as “idiopathic”. In these patients, diagnosis is still based on diagnostic criteria, but the etiology stands unknown even after specific clinical and extensive genetic evaluation [13].

## 4. Definition of ALVC: A Comparison with the 2020 Padua Criteria and the 2023 ESC Guidelines for the Management of Cardiomyopathies

The primary difference between the 2020 Padua criteria and the 2023 European TF criteria lies in the definition of ALVC. According to the 2020 Padua criteria, ALVC could only be diagnosed with positive genetic testing, thereby excluding conditions characterized by non-ischemic LV scarring and VAs secondary to other etiologies (e.g., post-myocarditis) or those that are idiopathic (negative genetic testing with no identifiable causes). For instance, a patient exhibiting an RBBB pattern and extensive subepicardial/midmyocardial late-enhancement on CMR, but no LV dilation/dysfunction and negative genetic testing, would paradoxically remain undiagnosed, even with a positive family history.

In contrast, the 2023 European TF criteria, by analogy with dilated and hypertrophic cardiomyopathies (which can be either primary or secondary), allow for the diagnosis of ALVC based on a combination of clinical criteria, regardless of the underlying cause. This approach highlights the significant risk of SCD associated with ALVC, irrespective of its etiology and even if the LV ejection fraction is relatively preserved. Further aligning with this new perspective, the authors of the European TF criteria have suggested renaming the disease from ACM to “scarring/arrhythmogenic cardiomyopathy”. The term “scarring” reflects the pathobiological basis of the disease across various etiologies, while “arrhythmogenic” describes the associated VAs, which are the main clinical manifestation and prognostic determinant.

In 2023, the European Society of Cardiology (ESC) proposed a new phenotypic classification of cardiomyopathies [44]. According to this classification, ACM should be diagnosed based on the 2010 ITF criteria, which do not include tissue characterization by CMR and require demonstration of RV abnormalities. Patients with ALVC would be categorized as either “dilated cardiomyopathy” or “non-dilated LV cardiomyopathy (NDLVC)”, based on end-diastolic LV volume. The umbrella term NDLVC encompasses patients with non-ischemic myocardial fibrosis with or without LV dysfunction (a definition that largely overlaps with ALVC), as well as those with LV dysfunction, no myocardial scarring, and no LV dilation. Therefore, the main distinction between the 2023 European TF criteria and the ESC guidelines is that the former primarily rely on tissue characterization, while the latter focus on morphological parameters.

We recognize the challenges in providing a simple classification for cardiomyopathies, diseases that can exhibit different phenotypic manifestations even among family members with the same genetic mutations. However, we believe that the definition of “scarring/arrhythmogenic cardiomyopathy”, identifying a disease characterized by non-ischemic fibrosis of the RV, LV, or both, and a high risk of VAs, is more accurate from a pathobiological standpoint and clinically more useful than the combination of the old definition of ARVC according to the 2010 ITF criteria plus the new definition of NDLVC that includes both patients with and without arrhythmogenic LV scarring.

## 5. Conclusions

The 2023 European TF criteria, updated from the Padua criteria, aim to improve diagnostic accuracy and precision focusing on LV involvement, which is often misdiagnosed or undiagnosed, ending up in considerable undertreatment (Tables 2,3).

**Table 2.** **Comparison between the 2020 International “Padua” Criteria and 2023 European Task Force criteria for diagnosis of Arrhythmogenic Cardiomyopathy**.

Category	2020 International criteria	2023 European Task Force criteria
1. Global or regional dysfunction and structural alteration	RV phenotype	RV phenotype
	*Major*	*Major*
	By 2D echocardiogram, CMR, or angiography:	• Regional RV akinesia, dyskinesia, or aneurysm plus one of the following:
	• Regional RV akinesia, dyskinesia, or bulging plus 1 of the following:	- global RV dilatation (increase of RV EDV according to the imaging test specific nomograms for age, sex and BSA)
	- global RV dilatation (increase of RV EDV according to the imaging test specific nomograms for age, sex, and BSA)	*or*
	*or*	- global RV systolic dysfunction (reduction of RV EF according to the imaging test specific nomograms for age and sex)
	- global RV systolic dysfunction (reduction of RV EF according to the imaging test specific nomograms for age and sex)	*Minor*
	*Minor*	• Regional RV akinesia, dyskinesia or aneurysm of RV free wall
	By 2D echocardiogram, CMR, or angiography:	
	• Regional RV akinesia, dyskinesia or aneurysm of RV free wall	
	LV phenotype	LV phenotype
	*Minor*	*Minor*
	By echocardiography, CMR or angiography:	• Global LV systolic dysfunction, with or without LV dilatation (increase of LV EDV according to the imaging test specific nomograms for age, sex, and BSA)
	• Global LV systolic dysfunction (depression of LV EF or reduction of echocardiographic global longitudinal strain), with or without LV dilatation (increase of LV EDV according to the imaging test specific nomograms for age, sex, and BSA)	
	*Minor*	
	• Regional LV hypokinesia or akinesia of LV free wall, septum, or both	
2. Tissue characterization	RV phenotype	RV phenotype
	*Major*	*Major*
	By CE-CMR:	• Fibrous replacement of the myocardium in ≥1 sample, with or without fatty tissue, at histology
	• Transmural LGE (stria pattern) of ≥1 RV region(s) (inlet, outlet, and apex in 2 orthogonal views)	*Minor*
	*Major*	• Unequivocal RV LGE (confirmed in 2 orthogonal views) in ≥1 RV region(s) (excluding tricuspid valve)
	By EMB (limited indications):	
	• Fibrous replacement of the myocardium in ≥1 sample, with or without fatty tissue	
	LV phenotype	LV phenotype
	*Major*	*Major*
	By CE-CMR	• “Ring-like” LV LGE (subepicardial or midmyocardial stria pattern) of ≥3 segments (confirmed in 2 orthogonal views)
	• LV LGE (stria pattern) of ≥1 Bull’s Eye segment(s) (in 2 orthogonal views) of the free wall (subepicardial or midmyocardial), septum, or both (excluding septal junctional LGE)	*Minor*
		• LV LGE (subepicardial or midmyocardial stria pattern) of 1 or 2 Bull’s Eye segment(s) (in 2 orthogonal views) of the free wall, septum, or both (excluding patchy, focal or septal junctional LGE)
3. Repolarization abnormalities	RV phenotype	RV phenotype
	*Major*	*Major*
	• Inverted T waves in right precordial leads (V1, V2, and V3) or beyond in individuals with complete pubertal development (in the absence of complete RBBB)	• Negative T waves in right precordial leads (V1, V2, and V3) or beyond in individuals ≥14-year-old (in the absence of complete RBBB and not preceded by J-point/ST-segment elevation)
	*Minor*	*Minor*
	• Inverted T waves in leads V1 and V2 in individuals with completed pubertal development (in the absence of complete RBBB)	• Negative T waves in leads V1 and V2 in males ≥14-year-old (in the absence of RBBB and not preceded by J-point/ST-segment elevation)
	• Inverted T waves in V1, V2, V3 and V4 in individuals with completed pubertal development in the presence of complete RBBB	• Negative T waves beyond V3 in the presence of complete RBBB
		• Negative T waves beyond V3 in individuals <14-year-old
	LV phenotype	LV phenotype
	*Minor*	*Minor*
	• Inverted T waves in left precordial leads (V4–V6) (in the absence of complete LBBB)	• Negative T waves in left precordial leads (V4–V6) (in the absence of complete LBBB)
4. Depolarization and conduction abnormalities	RV phenotype	RV phenotype
	*Minor*	*Minor*
	• Epsilon wave (reproducible low-amplitude signals between end of QRS complex to onset of the T wave) in the right precordial leads (V1 to V3)	• Epsilon wave (reproducible low-amplitude signals between end of QRS complex to onset of the T wave) in the right precordial leads (V1 to V3)
	• Terminal activation duration of QRS ≥55 ms measured from the nadir of the S wave to the end of the QRS, including R’, in V1, V2, or V3 (in the absence of complete RBBB)	• Terminal activation duration of QRS ≥55 ms measured from the nadir of the S wave to the end of the QRS, including R’, in V1, V2, or V3 (in the absence of complete RBBB)
	LV Phenotype	LV Phenotype
	*Minor*	*Major*
	• Low QRS voltages (<0.5 mV peak to peak) in limb leads (in the absence of obesity, emphysema, or pericardial effusion)	• Low QRS voltages (<0.5 mV peak to peak) in all limbs leads in the absence of other causes (e.g., cardiac amyloidosis, obesity, emphysema, or pericardial effusion)
5. Arrhythmias	RV Phenotype	RV Phenotype
	*Major*	*Major*
	• Frequent ventricular extrasystoles (>500 per 24 h), non-sustained or sustained ventricular tachycardia of LBBB morphology	• Frequent ventricular extrasystoles (>500 per 24 h), non-sustained or sustained ventricular tachycardia of LBBB morphology with non-inferior axis
	*Minor*	*Minor*
	• Frequent ventricular extrasystoles (>500 per 24 h), non-sustained or sustained ventricular tachycardia of LBBB morphology with inferior axis (“RVOT pattern”)	• Frequent ventricular extrasystoles (>500 per 24 h), non-sustained or sustained ventricular tachycardia of LBBB morphology with inferior axis (“RVOT pattern”)
		• History of cardiac arrest due to ventricular fibrillation or sustained ventricular tachycardia of unknown morphology
	LV phenotype	LV phenotype
	*Minor*	*Minor*
	• Frequent ventricular extrasystoles (>500 per 24 h), non-sustained or sustained ventricular tachycardia with a RBBB morphology (excluding the “fascicular pattern”)	• Frequent (>500 per 24 h) or exercise-induced ventricular extrasystoles with a RBBB morphology or multiple RBBB morphologies (excluding the “fascicular pattern”)
		• Non-sustained or sustained ventricular tachycardia with a RBBB morphology (excluding the “fascicular pattern”)
		• History of cardiac arrest due to ventricular fibrillation or sustained ventricular tachycardia of unknown morphology
6. Family history/genetics	RV/LV phenotype	RV/LV phenotype
	*Major*	*Major*
	• Identification of a pathogenic or likely pathogenetic ACM mutation in the patient under evaluation	• Identification of a pathogenic ACM-gene variant in the patient under evaluation
	• ACM confirmed in a first-degree relative who meets diagnostic criteria	• ACM confirmed in a first-degree relative who meets diagnostic criteria
	• ACM confirmed pathologically at autopsy or surgery in a first-degree relative	• ACM confirmed pathologically at autopsy or surgery in a first-degree relative
	*Minor*	*Minor*
	• History of ACM in a first-degree relative in whom it is not possible or practical to determine whether the family member meets diagnostic criteria	• Identification of a likely-pathogenic ACM-gene variant in the patient under evaluation
	• Premature sudden death (<35 years of age) due to suspected ACM in a first-degree relative	• History of ACM in a first-degree relative in whom it is not possible or practical to determine whether the family member meets diagnostic criteria
	• ACM confirmed pathologically or by diagnostic criteria in second-degree relative	• Premature sudden death (<35 years of age) due to suspected ACM in a first-degree relative
		• ACM confirmed pathologically or by diagnostic criteria in second-degree relative

ACM, arrhythmogenic cardiomyopathy; BSA, body surface area; CE-CMR, contrast enhanced cardiac magnetic resonance; EDV, end diastolic volume; EF, ejection fraction; EMB, endomyocardial biopsy; LBBB, left bundle-branch block; LGE, late gadolinium enhancement; LV, left ventricle; RBBB, right bundle-branch block; RV, right ventricle; RVOT, right ventricular outflow tract; 2D, 2-dimensional.

**Table 3.** **Summary of main updates of 2023 European Task Force Criteria compared to 2020 Padua Criteria**.

Category	2023 TF updates
1. Global or regional dysfunction and structural alteration	*LV*: the 2nd *Minor criterion* “Regional LV hypokinesia or akinesia of LV free wall, septum, or both” has been removed.
2. Tissue characterization	*RV*: now the specific *Major criterion* including histologic analysis after EMB is not a limited indication anymore; transmural LGE (non-ischemic pattern) at CMR is now considered as a *Minor criterion*.
	*LV: *added a new* Major criterion *about “Ring-like” LV LGE stria pattern of 3 or more segments; bull’s eye segments LGE involvement has been better defined and is now considered as a *Minor criterion*.
3. Repolarization abnormalities	*RV*: *Major criterion* has been better defined (complete pubertal development is now specified as ≥14-year-old and T wave must not be preceded by J-point/ST-segment elevation); now the old *Minor criteria* about negative T-wave beyond V3 have been split to include either presence of complete RBBB or ≥14-year-old individuals.
4. Depolarization and conduction abnormalities	*LV*: low QRS voltages criterion is now considered ad *Major* and has been better defined, specifying the need of exclusion all the other causes of low voltages.
5. Arrhythmias	*RV*: among *Minor criteria*, added “History of cardiac arrest due to ventricular fibrillation or sustained ventricular tachycardia of unknown morphology”.
	*LV*: relevance is given to exercise-induced ventricular extrasystoles to better define the first *Minor criteria*; added two new *Minor Criteria* about non-sustained or sustained ventricular tachycardia with a RBBB morphology (excluding the “fascicular pattern”) and history of cardiac arrest due to ventricular fibrillation or sustained ventricular tachycardia of unknown morphology.
6. Family history/genetics	*RV/LV*: added the identification of a likely-pathogenic ACM-gene variant in the patient under evaluation as a *Minor criterion*.

ACM, arrhythmogenic cardiomyopathy; CMR, cardiac magnetic resonance; EMB, endomyocardial biopsy; LGE, late gadolinium enhancement; LV, left ventricle; RBBB, right bundle-branch block; RV, right ventricle; TF, Task Force.

This review highlights and underlines the limitations of the 2010 ITF criteria, emphasizing the necessity of a diagnosis improvement for ACM and giving a practical list of “Six categories criteria”, as a useful modified expansion of the 2020 Padua criteria. Moreover, the use of the new definition “scarring/arrhythmogenic cardiomyopathy” in describing this condition, allows for targeting of the characteristic non-ischemic myocardial scar together with arrhythmic predisposition regardless of the etiology.

Clinical and diagnostic evolution make us confident about the practical use of this criteria, however we strongly believe that “every-day” clinical application of this 2023 update is crucial for their validation, with the goal of accelerating and improving the diagnosis and treatment of ACM.

## Abbreviations

ACM, arrhythmogenic cardiomyopathy; BSA, body surface area; CMR, cardiac magnetic resonance; EDV, end diastolic volume; EF, ejection fraction; EMB, endomyocardial biopsy; ITF, International Task Force; LBBB, left bundle-branch block; LGE, late gadolinium enhancement; LV, left ventricle; RBBB, right bundle-branch block; RV, right ventricle; RVOT, right ventricular outflow tract.
